# On multi-path longitudinal spin relaxation in brain tissue

**Published:** 2023-01-20

**Authors:** Jakob Assländer, Andrew Mao, Erin S Beck, Francesco La Rosa, Robert W Charlson, Timothy M Shepherd, Sebastian Flassbeck

**Affiliations:** a Center for Biomedical Imaging, Dept. of Radiology, New York University School of Medicine, 650 1st Avenue, New York, 10016, NY, USA; b Center for Advanced Imaging Innovation and Research (CAI2R), Dept. of Radiology, New York University School of Medicine, 650 1st Avenue, New York, 10016, NY, USA; c Vilcek Institute of Graduate Biomedical Sciences, New York University School of Medicine, 550 1st Avenue, New York, 10016, NY, USA; d Corinne Goldsmith Dickinson Center for Multiple Sclerosis, Department of Neurology, Icahn School of Medicine at Mount Sinai, 5 East 98th Street, New York, 10029, NY, USA; e Department of Neurology, New York University School of Medicine, 240 E 38th Street, New York, 10016, NY, USA

**Keywords:** quantitative MRI, qMRI, parameter mapping, relaxometry, MR Fingerprinting, Multiple Sclerosis

## Abstract

The purpose of this paper is to confirm previous reports that identified magnetization transfer (MT) as an inherent driver of longitudinal relaxation in brain tissue by asserting a substantial difference between the *T*_1_ relaxation times of the *free* and the *semi-solid spin pools*. Further, we aim to identify an avenue towards the quantification of these relaxation processes on a voxel-by-voxel basis in a clinical imaging setting, i.e. with a nominal resolution of 1mm isotropic and full brain coverage in 12min. To this end, we optimized a *hybrid-state* pulse sequence for mapping the parameters of an unconstrained MT model. We scanned 4 people with relapsing-remitting multiple sclerosis (MS) and 4 healthy controls with this pulse sequence and estimated $T_{1}^{f}\approx 1.90\text{s}$ and $T_{1}^{s}\approx 0.327\text{s}$ for the free and semi-solid spin pool of healthy WM, respectively, confirming previous reports and questioning the commonly used assumptions $T_{1}^{s}=T_{1}^{f}$ or $T_{1}^{s}=1\text{s}$. Further, we estimated a fractional size of the semi-solid spin pool of $m_{0}^{s}\approx 0.202$, which is larger than previously assumed. An analysis of $T_{1}^{f}$ in normal appearing white matter revealed statistically significant differences between individuals with MS and controls. In conclusion, we confirm that longitudinal spin relaxation in brain tissue is dominated by MT and that the hybrid state facilitates a voxel-wise fit of the unconstrained MT model, which enables the analysis of subtle neurodegeneration.

## 1. Introduction

Longitudinal relaxation is an important contrast mechanism in magnetic resonance imaging (MRI). E.g., the MP-RAGE (Mugler and Brookeman, 1990) pulse sequence generates excellent gray matter (GM) - white matter (WM) contrast and, compared to mostly *T*_2_-weighted pulse sequences like FLAIR (Hajnal et al., 1992), may be more specific to the underlying tissue changes in multiple sclerosis (MS) lesions (Barkhof, 1999; Bagnato et al., 2003).

Quantitative assessment of longitudinal relaxation could improve inter-scan, -scanner, and -subject comparability. Most commonly, such quantification has been based on a mono-exponential fit of the recovery to thermal equilibrium governed by the time constant *T*_1_ (*T*_1_ ≈ 1.084s in WM at 3T (Stanisz et al., 2005)). This relaxation model for biological tissue is adopted from the theoretically well-founded mono-exponential relaxation model for liquids (Bloch, 1946; Bloembergen et al., 1948). A key advantage of this model is its simplicity and the ease of measuring *T*_1_. However, mono-exponential *T*_1_-mapping of brain white matter is inconsistent due to substantial inter-sequence and inter-scanner variability (Stikov et al., 2015; Bojorquez et al., 2016). A potential source of this variability is the complexity of the relaxation mechanisms in biological tissue that are not captured by a mono-exponential model. Recent studies (Gochberg and Gore, 2003; Helms and Hagberg, 2009; Gelderen et al., 2016; Manning et al., 2021; Samsonov and Field, 2021) suggest that magnetization transfer (MT) (Wolff and Balaban, 1989; Henkelman et al., 1993) is a key contributor to the observed longitudinal relaxation in WM.

Magnetization transfer is commonly described by Henkelman’s two-pool model (Henkelman et al., 1993), where one spin pool, the *free* pool, consists of all protons bound in water and the other pool, the *semi-solid* pool, consists of protons bound in macromolecules such as proteins and lipids. In standard clinical pulse sequences, one does not observe the latter spins directly since their transversal magnetization relaxes below the noise level before we can observe it $\left(T_{2}^{s}\approx 10\mu \text{s}\right)$. However, transfer of longitudinal magnetization between the two pools alters the free pool’s longitudinal spin relaxation in biological tissue. RF-pulses inherently modify the semi-solid pool’s longitudinal magnetization and, hence, alter the relaxation of the free pool.

The indirect nature of MT complicates the estimation of the model’s parameters. $T_{1}^{s}$, in particular, is difficult to estimate and the vast majority of quantitative MT (qMT) studies assume either $T_{1}^{s}=1\text{s}$ (Henkelman et al., 1993; Morrison and Henkelman, 1995) or $T_{1}^{s}=T_{1}^{f}\approx 1.1\text{s}$ (Yarnykh, 2002; Dortch et al., 2011). However, more recent studies have suggested that $T_{1}^{s}\approx 0.3\text{s}$ and $T_{1}^{f}\approx 2\text{s}$ (Helms and Hagberg, 2009; Gelderen et al., 2016; Manning et al., 2021; Samsonov and Field, 2021). These estimates suggest that, contrary to common assumptions, MT is not a mechanism that has to be emphasized with dedicated saturation pulses, but rather suggest that MT is an inherent driver of longitudinal relaxation. The first goal of this paper is to confirm these findings.

Due to the difficulty of estimating $T_{1}^{s}$, previous approaches have used brain-wide estimates of $T_{1}^{s}$ and/or $T_{1}^{f}$ (Gelderen et al., 2016; Samsonov and Field, 2021) or fit the MT model to NMR samples (Manning et al., 2021) or a single large ROI averaged over multiple subjects (Helms and Hagberg, 2009). The second goal of this paper is to enable a voxel-wise estimation of the unconstrained two-pool MT model. Key to this advance is a *hybrid state* (Assländer et al., 2019b) of the free spin pool that can provide increased efficiency in the encoding and the disentanglement of the MT and relaxation processes (Assländer, 2021). Further, we describe the semi-solid spin pool with the *generalized Bloch model* for slight improvements in model accuracy (Assländer et al., 2022a). With this approach, we are able to perform unconstrained qMT imaging with a clinically-established resolution (1mm isotropic as measured by the maximum k-space frequency) and a scan time of 12 minutes.

## 2. Methods

### 2.1. Magnetization Transfer Model

We use the MT model described in Assländer et al. (2022a, b), which builds on Henkelman’s two-pool spin model (Henkelman et al., 1993) and captures the two pools with a Bloch-McConnell equation (McConnell, 1958):

(1) $$\partial_{t}\left(\begin{array}{c} x^{f}\\ y^{f}\\ z^{f}\\ x^{s}\\ z^{s}\\ 1 \end{array}\right)=\left(\begin{array}{cccccc} -R_{2}^{f} & -\omega_{z} & \omega_{y} & 0 & 0 & 0\\ \omega_{z} & -R_{2}^{f} & 0 & 0 & 0 & 0\\ -\omega_{y} & 0 & -R_{1}^{f}-R_{\text{x}}m_{0}^{s} & 0 & R_{\text{x}}m_{0}^{f} & m_{0}^{f}R_{1}^{f}\\ 0 & 0 & 0 & -R_{2}^{s,l}\left(R_{2}^{s},\alpha ,T_{\text{RF}}\right) & \omega_{y} & 0\\ 0 & 0 & R_{\text{x}}m_{0}^{s} & -\omega_{y} & -R_{1}^{s}-R_{\text{x}}m_{0}^{f} & m_{0}^{s}R_{1}^{s}\\ 0 & 0 & 0 & 0 & 0 & 0 \end{array}\right)\left(\begin{array}{c} x^{f}\\ y^{f}\\ z^{f}\\ x^{s}\\ z^{s}\\ 1 \end{array}\right).$$

**Figure 1:**
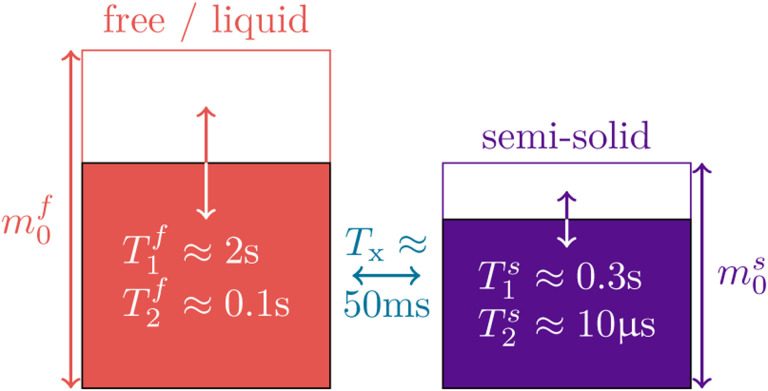
Sketch of the two-pool magnetization transfer model (Henkelman et al., 1993). This model jointly describes all magnetization arising from protons bound in liquids by the spin pool $m_{0}^{f}$; and all magnetization arising from protons bound in macromolecules by the pool $m_{0}^{s}$ whose transversal relaxation time is several orders of magnitude shorter. We normalize the thermal equilibrium magnetization to $m_{0}^{f}+m_{0}^{s}=1$ and describe the magnetization transfer between the pools by the rate *R*_x_ = 1*/T*_x_. The model is governed by Eq. (1).

The *free* pool, sketched in red in Fig. 1, captures all protons bound in liquids where fast molecular motion causes an exponential relaxation of the transversal magnetization with a characteristic $T_{2}^{f}≳50\text{ms}$ (Bloembergen et al., 1948). The free pool’s magnetization is described by the Cartesian coordinates *x*^*f*^, *y*^*f*^, *z*^*f*^, the off-resonance frequency is described by *ω*_*z*_, and the Rabi frequency of the RF pulses by *ω*_*y*_. For readability, we here use relaxation rates $\left(R_{1,2}^{f,s}=1/T_{1,2}^{f,s}\right)$. The magnetization components *x*^*s*^,* z*^*s*^ of the *semi-solid* spin pool, sketched in purple in Fig. 1, capture all protons bound in large molecules such as lipids. The motion of such molecules is restricted, resulting in a much faster and non-exponential relaxation with a characteristic time constant of $T_{2}^{s}\approx 10\mu \text{s}$. For the here-examined brain tissue, we assume the decay characteristics associated with a super-Lorentzian lineshape (Morrison and Henkelman, 1995). The non-exponential characteristics of this lineshape prohibit a description with the original Bloch equations but such dynamics can be described with the *generalized Bloch model* (Assländer et al., 2022a). In Eq. (1), the generalized Bloch model is captured in its *linearized* form by the relaxation rate $R_{2}^{s,l}\left(R_{2}^{s},\alpha ,T_{\text{RF}}\right)$ that depends, in addition to the biophysical parameter $R_{2}^{s}$, on the flip angle *α* and the duration *T*_RF_ of respective RF-pulse. We neglect the *y*^*s*^ component assuming, without loss of generality, *ω*_*x*_ = 0 and given that $R_{2}^{s,l}\gg \omega_{z}$. Exchange processes between the pools are captured by the exchange rate *R*_x_. A sixth dimension is added to allow for a compact notation of the longitudinal relaxation to a non-zero thermal equilibrium.

### 2.2. Pulse sequence design

As mentioned above, we utilize the hybrid state (Assländer et al., 2019b) and its flexibility to encode and disentangle the different relaxation mechanisms. Like in balanced SSFP sequences (Carr, 1958), we balance all gradient moments in each *T*_R_. Unlike in SSFP sequences, we vary the flip angle and the duration of the RF-pulses. During slow flip angle variations, the direction of the magnetization establishes a steady state and adiabatically transitions between the steady states associated with different flip angles. As we showed in Assländer et al. (2019b), moderate change rates of the flip angle simultaneously yield a transient state of the magnetization’s magnitude and we call this combination the *hybrid state*. It combines the tractable off-resonance characteristics of the bSSFP sequence, in particular the refocusing of intra-voxel dephasing (Carr, 1958; Scheffler and Hennig, 2003), with the encoding potential of the transient state.

Our pulse sequence consists of a rectangular *π* inversion pulse, flanked by crusher gradients, and followed by a train of rectangular RF-pulses with varying flip angles and pulse durations, and with a *π* phase increment between consecutive RF pulses. The pulses are separated by a *T*_R_ = 3.5ms, which is approximately the minimal *T*_R_ with which we can perform gradient encoding with 1mm isotropic resolution and avoid stimulating the peripheral nerves. After 1142 RF-pulses, i.e. after a *cycle time* of 4s, the remaining magnetization is inverted by the next *π* pulse, then the same pulse train is repeated.

The relaxation and MT processes are encoded with two established mechanisms: first, the inversion pulse inverts the free pool and keeps the semi-solid pool largely unaffected. As described by Gochberg and Gore (2003) this induces a bi-exponential inversion recovery curve of the free pool composed of its intrinsic longitudinal relaxation and cross relaxation to the semi-solid spin pool. Second, the flip angle and the pulse durations can be used to control the different relaxation paths. In good approximation, the RF-pulse duration only affects the saturation of the semi-solid spin pool’s longitudinal magnetization (Gloor et al., 2008). In contrast, changes in the flip angle affect the relaxation processes of the free pool (Assländer et al., 2019a,b), the magnetization transfer between the two pools, and the saturation of the semi-solid spin pool (Gloor et al., 2008). More details on this interplay can be found in Assländer et al. (2022b).

### 2.3. Numerical optimization of the pulse train

On the basis of these two encoding mechanisms, we numerically optimized the flip angles and pulse durations of RF-pulse trains. The optimization objective was the Cramér-Rao bound (CRB) (Rao, 1945; Cramér, 1946) of the relaxation rates and the other model parameters and was calculated as described in Assländer et al. (2022b). We optimized a separate pulse train for each of the biophysical parameters $m_{0}^{s}$, $R_{1}^{f}$, $R_{2}^{f}$, *R*_x_, $R_{1}^{s}$, and $T_{2}^{s}$, while, additionally accounting for *ω*_*z*_, $B_{1}=\omega_{y}/\omega_{y}^{\text{nominal }}$, and the scaling factor *M*_0_ as unknowns. Additionally, we optimized a pulse train for the sum of the CRBs of all biophysical parameters, normalized with respective squared parameter values to resemble the inverse squared SNR. All simulations and CRB calculations were performed with $m_{0}^{s}=0.25$, $R_{1}^{f}=0.5/\text{s}$, $R_{2}^{f}=15.4/\text{s}$, *R*_x_ = 20/s, $R_{1}^{s}=2/\text{s}$, $T_{2}^{s}=10\mu \text{s}$, and *ω*_*z*_ = 0, and *B*_1_ = 1.

### 2.4. Phantom scan

We built a custom phantom composed of cylindrical 50mL tubes filled with different concentrations of thermally cross-linked bovine serum albumin (BSA). We mixed the BSA powder (10%, 15%, and 20% of the total weight) with distilled water and stirred it at 30°C until the BSA was fully dissolved. We divided the solution in half and added 0.1mM MnCl_2_ to one half. We filled 6 tubes with the resulting solutions and thermally cross-linked them in a water bath at approximately 90°C for 10 minutes. All tubes were embedded in a container filled with distilled water and 0.1mM MnCl_2_.

We scanned this phantom on a 3T Prisma scanner (Siemens, Erlangen, Germany) using either a 32-channel head coil. We performed a 6min scan with each of 6 individual optimizations, resulting in a 36min overall scan time. For each 6min scan, the RF-pattern is repeated 90 times during which we acquire 3D radial k-space spokes with nominal 1mm isotropic resolution (defined by |*k*_max_| = *π/*1mm). We changed the direction of the k-space spokes with a 2D golden means pattern (Winkelmann et al., 2007; Chan et al., 2009) that is reshuffled to improve the k-space coverage for each time point and to minimize eddy current artifacts (Flassbeck and Assländer, 2022).

### 2.5. *In vivo* scans

In order to establish high-quality reference data, we performed in vivo scans of 4 individuals with clinically-established relapsing-remitting MS (age 37.5 ± 8.7, 3 female) and 4 healthy controls (age 28.8±5.6, 3 female) with the 36min protocol described in Section 2.4. In addition to the hybrid-state scans, we performed 3D MP-RAGE and FLAIR scans, also each with 1mm isotropic resolution.

To test more clinically feasible scan times, we scanned an additional MS patient with three protocols:

- 1.0mm isotropic in 12min
- 1.3mm isotropic in 6min
- 1.6mm isotropic in 4min.

### 2.6. Image reconstruction

For the 36min reference scans, each of the 6 scans, we reconstructed 13 coefficient images in the low-dimensional space spanned by singular vectors from a coarse dictionary of signals (or fingerprints) (McGivney et al., 2014; Tamir et al., 2017; Assländer et al., 2018). We used the FISTA algorithm (Coyne et al., 2009), incorporating sensitivity encoding (Sodickson and Manning, 1997; Pruessmann et al., 2001) and locally low-rank regularization (Lustig et al., 2007; Trzasko and Manduca, 2011; Zhang et al., 2015) to reduce residual undersampling artifacts and noise. We implemented this reconstruction in *Julia* and made the source code publicly available (cf. Appendix A). A more detailed description of the reconstruction can be found in Tamir et al. (2017) and Assländer et al. (2018). Iterscan motion was corrected by applying a rigid registration to the first coefficient of scan 2–6 to the first scan using Freesurfer (“mri_robust_register”) (Reuter et al., 2010). The respective transformation matrices were subsequently applied to transform each set of coefficient images onto the same grid using trilinear interpolation.

For phantom scan and the rapid protocols, we reconstructed all data of the 6 sub-scans into a joint 15-dimensional subspace with otherwise identical settings.

### 2.7. Model fitting

For computational efficiency and robustness, we used a neural network (NN) to fit the relaxation/MT model, including a data-driven *B*_0_ and *B*_1_ correction (Assländer et al., 2022b), voxel by voxel to the reconstructed coefficient images from all 6 scans jointly (Cohen et al., 2018; Nataraj et al., 2018; Duchemin et al., 2020; Zhang et al., 2022). Our network, implemented using the *Flux.jl* package, closely follows the design described in Fig. 2 of Zhang et al. (2022), retaining a similar overall architecture: the input vector (6×13 or 15 complex valued coefficients for the two different version of the image reconstruction, normalized by the first coefficient and then split into real and imaginary part) are up-sampled to size 1024 before down-sampling again over 8 fully connected layers with skip connections. The NN outputs estimates of all 6 unconstrained MT parameters, where each individual parameter is constrained with a ReLU function capped at the maximum value expected *in vivo*. We trained the NN for 750 epochs using the Rectified ADAM optimizer (Liu et al., 2019) with a learning rate of 10^−3^ and an inverse time decay rate of 5·10^−4^. For comparison, we separately trained NNs to fit the Bloch model and MT models that are constrained to $T_{1}^{s}=T_{1}^{f}$ or $T_{1}^{s}=1\text{s}$.

### 2.8. Region of interest analysis

For the 36min reference scans, we registered the MP-RAGE and the FLAIR images to the brain-masked (Hoopes et al., 2022) qMT maps with the FreeSurfer package (“mri_robust_register”) (Reuter et al., 2010). We also used FreeSurfer (“recon-all”) to segment the brain based on the MP-RAGE and the FLAIR (Fischl et al., 2002, 2004). We extracted region of interest (ROI) masks for the entire normal appearing white matter (NAWM), several WM subregions, the cortical GM, and subcortical GM structures. To ensure that MS lesions were excluded from the ROIs, we calculated lesion masks with an in-house developed deep learning model, based on the nnUNet framework (Isensee et al., 2021) using the FLAIR images. The automated lesion segmentations were manually adjusted by FLR and ESB and subtracted from the ROI masks. Thereafter, we eroded the outmost layer of voxels of each ROI to reduce partial volume effects with other tissues and to ensure that all ROI voxels are at least one voxel away from the next lesion.

## 3. Results

### 3.1. Numerical optimizations of the pulse train

**Figure 2:**
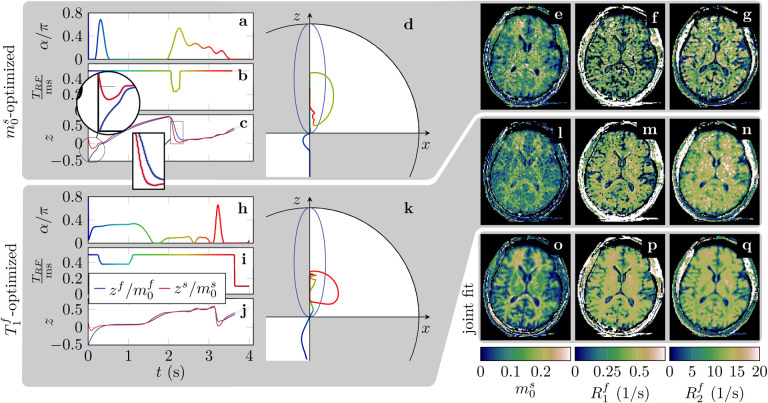
Optimized RF-pulse trains, evoked spin dynamics, and corresponding in vivo parameter maps. **a,h** The flip angle *α* and **b,i** the pulse duration *T*_RF_ control the spin dynamics. **c,j** The normalized *z*-magnetization of the two pools. The spherical and rectangular magnifications in (c) highlight segments that utilize a bi-exponential inversion-recovery (Gochberg and Gore, 2003) and a saturation of the semi-solid spin pool (Gloor et al., 2008), respectively, which encode the semi-solid pool size $m_{0}^{s}$. **d,k** The dynamics of the free pool on the Bloch sphere with the steady-state ellipse in blue. **e-g** $m_{0}^{s}$ and the relaxation times of the free pool $T_{1}^{f}$ and $T_{2}^{f}$ maps that were estimated from a 6min scan with an $m_{0}^{s}$-optimized RF-pattern in comparison to **l-n** a pattern that was optimized for $T_{1}^{f}$ and **o-q** a joint fit of 6 measurements with RF-patterns that were optimized for different qMT parameters (36min scan time).

The numerical optimizations of the pulse train resulted in smooth flip angle and *T*_RF_ patterns. Fig. 2 sketches the RF-pattern that were optimized for $m_{0}^{s}$ and $R_{1}^{f}$, respectively, along with the evoked spin trajectories. We observe distinct patterns for the different optimization objectives (Fig. 2) that correspond to distinct CRB values (Tab. 1). To give one example, optimizing for $m_{0}^{s}$ resulted in a normalized CRB of 47s for $m_{0}^{s}$ and of 3837s for $R_{1}^{f}$, while the optimization for $R_{1}^{f}$ resulted in a normalized CRB of 2649s for $m_{0}^{s}$ and of 91s for $R_{1}^{f}$. This difference in CRB values is in line with the noise levels in scans with each of the two RF patterns: the optimization for $m_{0}^{s}$ results in a comparably low noise level in $m_{0}^{s}$ (Fig. 2e) and a comparably high noise level in $R_{1}^{f}$ (f), while the optimization for $R_{1}^{f}$ results in the opposite noise characteristics (l vs. m).

As the optimizations aim to disentangle the effect of 9 overall parameters, the resulting spin trajectories are difficult to interpret. Nonetheless, we can discern some features: for example, the $m_{0}^{s}$-optimized pattern starts with near-zero flip angles after the inversion pulse, which provokes a bi-exponential inversion recovery of the longitudinal magnetization (circular magnification in Fig. 2c) that encodes $m_{0}^{s}$ similar to the *SIR* method proposed by Gochberg and Gore (2003). The rectangular magnification highlights a section of the spin dynamics in which large flip angles, combined with short *T*_RF_, saturate the semi-solid spin pool, which resembles the encoding mechanism proposed by Gloor et al. (2008). This saturation maximizes the difference between pools, and this encoding mechanism is not as pronounced in the spin trajectory evoked by the $R_{1}^{f}$-optimized pattern, where the semi-solid spin pool plays a subordinate role.

Concatenating the 6 individual optimizations for $m_{0}^{s}$, $R_{1}^{f}$, $R_{2}^{f}$, *R*_x_, $R_{1}^{s}$, and $\;T_{2}^{s}$, respectively, increases the CRB values (normalized by the scan time) compared to the optimized CRB value of each specialized RF-pattern, but provides overall low CRB values for all six parameters that also results in high quality parameter maps (Fig. 2o–q). Interestingly, these CRB values are consistently lower than a joint optimization for all parameters. We note that the latter has to encode all parameters in a single 4s long cycle, while the former utilizes 6 distinct RF patters and has, thus, more degrees of freedom to induce different spin dynamics. Given this result, we performed all experiments with concatenated scans that utilize the 6 individual optimizations.

### 3.2. Phantom scan

**Figure 3:**
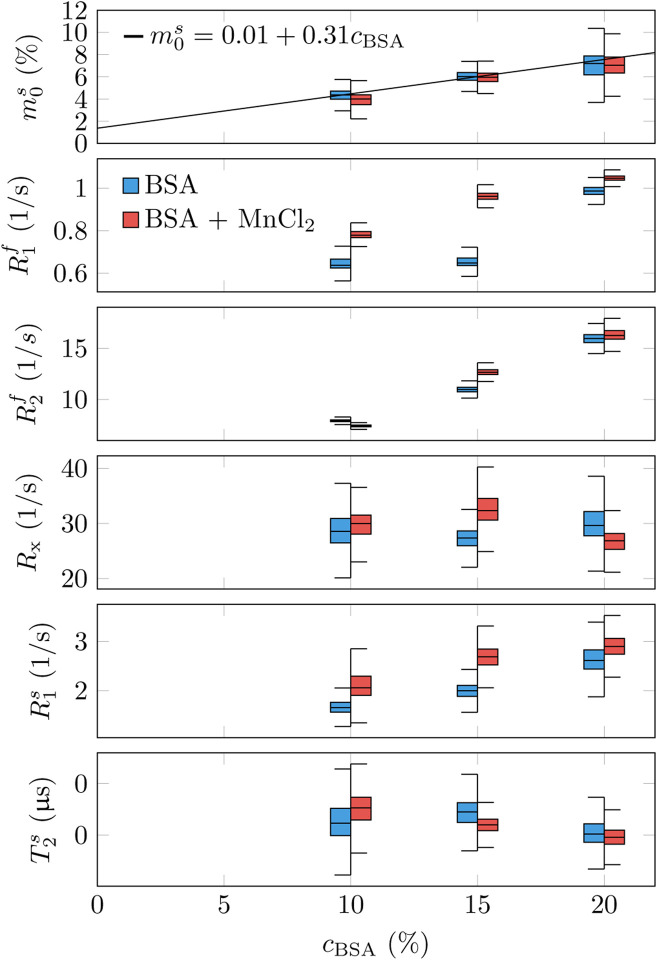
Phantom Validation. Six tubes filled with different concentrations of bovine serum albumin (BSA) and half of them dopped with 0.1mM MnCl_2_ were immersed in water-filled container and imaged. The box plots represent median, 1^st^ and 3^rd^ quartile, and the whiskers the 1.5x the inter-quartile range, limited by the maximum range of the voxel’s data. The mean values of each tube’s $m_{0}^{s}$ estimates was fitted with linear regression.

Due to limited number of prior work with the here described unconstrained qMT model and the experimental complexity of these approaches, we limit the analysis of the phantom scan to a comparison to a chemical ground truth, i.e., we compare the qMT estimates of each tube to their BSA and MnCl_2_ concentration (Fig. 3). We observe a linear dependency of $m_{0}^{s}$ and $R_{2}^{f}$ and, to a lesser degree, of $R_{1}^{f}$ on the BSA concentration, while the exchange rate and the semi-solid spin pool’s relaxation times show little-to-no variation with the BSA concentration. Doping the samples with MnCl_2_ has a strong effect on $R_{1}^{f}$ and a limited effect on all other parameters, with the outlier of the $R_{2}^{f}$ estimate in the 15% BSA sample.

We performed linear regression of each tube’s mean $m_{0}^{s}$ estimates as a function of their BSA concentrations while treating the samples with and w/o MnCl_2_ doping as independent samples. The linear model fits the data well and the intercept with the y-axis is at $m_{0}^{s}=0.0114\pm 0.0044$, i.e. it differs only slightly from the anticipated zero intercept. The slope of the linear regression is 0.310 ± 0.028. Assuming the chemical structure C_123_H_193_N_35_O_37_ for BSA and calculating, in approximation, the molecular weight simply by adding each atom’s weight, we can compare the number of protons per weight in BSA to the one in water. Based on this approximation, we expect a linear dependency with $m_{0}^{s}=0.63c_{\text{BSA}}$. By comparing to the measured $m_{0}^{s}=0.0114+0.310c_{\text{BSA}}$, we can estimate that roughly 50% of protons in BSA contribute to the MT effect, assuming all water protons contribute to the signal, i.e., assuming similar Boltzmann distributions for the two pools.

### 3.3. Reference scans of healthy volunteers

**Figure 4:**
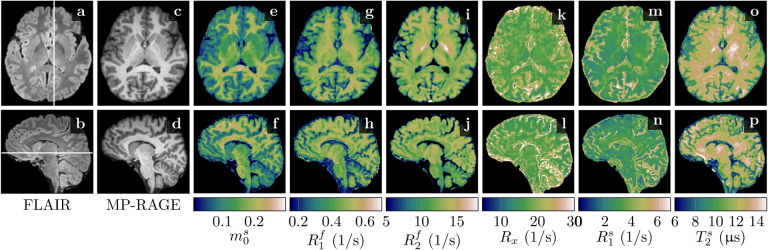
Comparison of clinical contrasts (**a**-**d**) and quantitative magnetization transfer (qMT) maps (**e**-**p**) in a healthy volunteer. All scans have a nominal resolution of 1mm isotropic and the qMT scan took 36min. We display here relaxation rates $\left(R_{1,2}^{f,s}=1/T_{1,2}^{f,s}\right)$, where the superscripts *f* and *s* indicate the *free* and *semi-solid* pools, respectively. The size of the semi-solid spin pool is normalized by $m_{0}^{s}+m_{0}^{f}=1$ and *R*_x_ denotes the exchange rate between the two pools.

Fig. 4 demonstrates the feasibility of unconstrained qMT imaging with a hybrid-state pulse sequence. We are able to encode all 6 biophysical parameters on a voxel-by-voxel basis with full brain coverage, a nominal resolution of 1mm isotropic and a scan time of 36min. We observe overall good image quality in $m_{0}^{s}$, $R_{1}^{f}$, and $R_{2}^{f}$, and slightly reduced image quality in *R*_x_, $R_{1}^{s}$, and $T_{2}^{s}$, consistent with the corresponding higher CRB values (Tab. 1). The $T_{2}^{s}$ map in particular is heterogeneous throughout the brain, which might, in part, be a residual *B*_1_ artifact. We also found subtle residual *B*_1_ artifacts in $R_{2}^{f}$ (Fig. 4i,o and Fig. 6b,f) and residual *B*_0_ artifacts in a few voxels at the center of the bSSFP banding artifact (Fig. 4f,h,… at the base of the frontal cortex). Beyond these residual artifacts, we found overall good performance of the *B*_0_ and *B*_1_ correction. The cerebellum reveals a slightly reduced effective resolution in comparison to the nominally equivalent resolution of the MP-RAGE (Fig. 4d vs. f,h,…).

Among all qMT parameters, we observe the largest quantitative GM/WM contrast in $m_{0}^{s}$, followed by $R_{1}^{f}$. In $R_{2}^{f}$, however, we observe only a subtle contrast between cortical GM and WM. This is confirmed by the ROI analysis that resulted in $T_{2}^{f}\approx (90.8\pm 6.4)\text{ms}$ and (79.6±6.8)ms for cortical GM and WM, respectively, which is a smaller difference compared to the difference between previously reported values (99 ± 7 vs. 69 ± 3ms) (Stanisz et al., 2005). We observed the shortest $T_{2}^{f}\approx (59.5\pm 2.3)\text{ms}$ in the globus pallidus (Fig. 4i). The exchange rate *R*_x_ is slightly larger in GM compared to WM ((19.8 ± 2.3)/s vs. (16.55 ± 0.99)/s), while $R_{1}^{s}$ and $T_{2}^{s}$ exhibit very little GM/WM contrast. We note that the most prominent contrast in *R*_x_, $R_{1}^{s}$ and $T_{2}^{s}$ occurs in voxels that are subject to partial volume effects and in CSF, where the small $m_{0}^{s}$ makes estimates of semi-solid spin-pool characteristics unreliable.

Estimates of the unconstrained MT model’s parameters are reported in Tab. 2 for several WM and GM structures. In the following, we will analyze the (entire) WM and the cortical GM in more detail and compare the estimates with the unconstrained MT model to the Bloch model and constrained MT models.

#### 3.3.1. White matter

**Figure 5:**
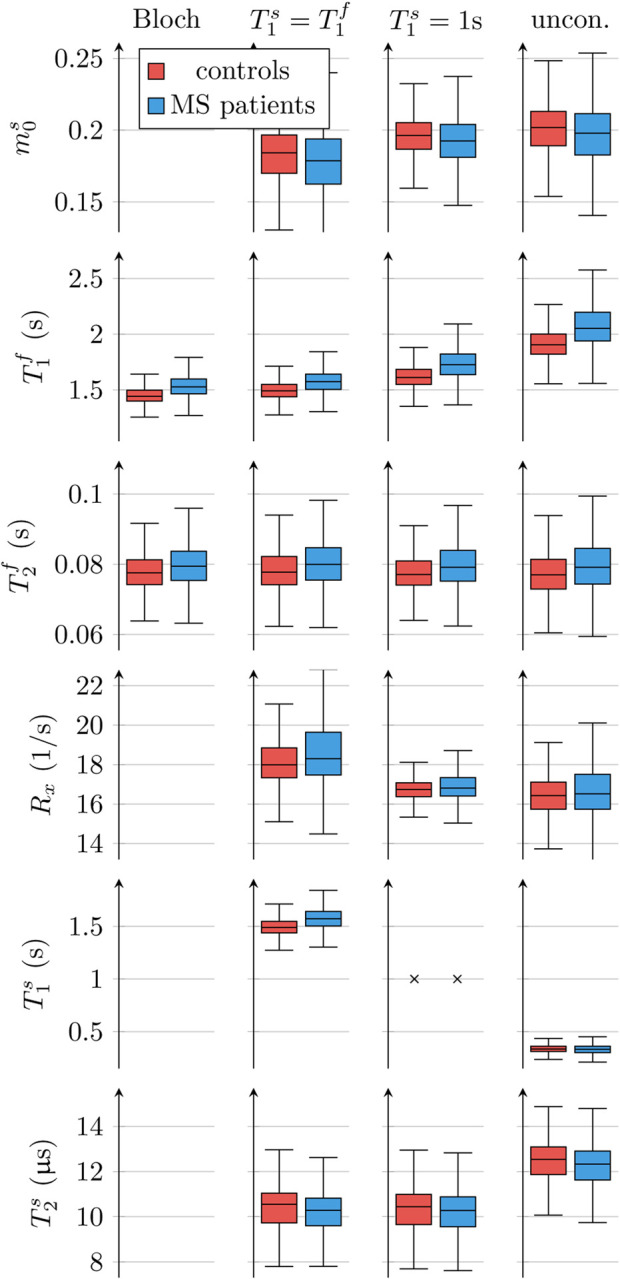
Comparison of the parameter estimates between a Bloch model, two traditional MT models that assume $T_{1}^{s}=T_{1}^{f}$ and $T_{1}^{s}=1\text{s}$, respectively, and the proposed unconstrained MT model. This analysis pools all normal appearing white matter voxels of 4 healthy subjects and 4 individuals with MS, respectively. Note that the $T_{1}^{s}=T_{1}^{f}$ column depicts the same longitudinal relaxation time estimates twice to illustrate the differences to the unconstrained MT model.

Tab. 2 and Fig. 5 display an ROI analysis of the entire white matter, averaged over all healthy volunteers. It confirms that $T_{1}^{f}\approx (1.90\pm 0.14)\text{s }$ and $T_{1}^{s}\approx (0.327\pm 0.033)\text{s}$, as estimated with the unconstrained MT model, differ substantially from one another and from mono-exponential or Blochian estimates from our data (1.429 ± 0.069)s and mono-exponential estimates reported in the literature ((1.084 ± 0.045)s (Stanisz et al., 2005)). We note that differences between different mono-exponential estimates are not surprising due to the oversimplified nature of this model (cf. Sections 1 and 4).

The estimated $m_{0}^{s}\approx 0.202\pm 0.018$ (using the unconstrained MT model) is in line with literature estimates that use the same model (0.172 ± 0.043; (Helms and Hagberg, 2009)), but larger than estimates with a constrained MT model (0.139 ± 0.028; (Stanisz et al., 2005) and 0.118 ± 0.050; (Helms and Hagberg, 2009)). We estimated 0.184 ± 0.020 from our data with the model that is constrained by $T_{1}^{s}=T_{1}^{f}$, which differs from literature estimates. Once again, deviations to estimates made with different models are not surprising and we note that constrained MT models consistently bias $m_{0}^{s}$ to smaller values compared to the unconstrained MT model.

The exchange rate *R*_x_ ≈ (16.55±0.99)/s, as estimated with the unconstrained MT model, also compares well with the literature that utilizes this model ((18.1±3.6)/s (Helms and Hagberg, 2009)), while it is lower compared to literature values that utilize a constrained MT model ((23±4)/s (Stanisz et al., 2005)). Our estimate of (18.3±1.2)/s with the MT model that is constrained by $T_{1}^{s}=T_{1}^{f}$ also differs from above literature estimates. However, the biases when using constrained models suggest faster exchange compared to the unconstrained model.

Our estimates of $T_{2}^{f}$ are largely independent of the model ((79.6 ± 6.8)ms with the unconstrained model, (79.9±6.2)ms with the constrained MT model, and (79.7± 5.7)ms with the Bloch model). However, these values are larger than literature values ((69 ± 3)ms (Stanisz et al., 2005)).

The estimated $T_{2}^{s}\approx (12.3\pm 1.0)\mu \text{s}$ (using the unconstrained MT model) is slightly larger than estimates using a constrained model ((10.42 ± 0.96)μs using our data or (11.3 ± 1.8)μs as estimated by Stanisz et al. (2005)).

#### 3.3.2. Gray matter

An ROI analysis of the cortical gray matter, averaged over all healthy volunteers, reveals trends similar to the WM analysis: $T_{1}^{f}\approx (2.89\pm 0.41)\text{s}$ and $T_{1}^{s}\approx (0.299\pm 0.074)\text{s}$ differ substantially from one another, from the mono-exponential estimate (1.82±0.11)s that was measured by Stanisz et al. (2005).

The estimated $m_{0}^{s}\approx 0.082\pm 0.014$ (using the unconstrained model) is, as expected, smaller than in WM. Further, it is larger than literature values that are based on a constrained MT model (0.050 ± 0.005 (Stanisz et al., 2005)). We were not able to find literature values for GM that use the unconstrained MT model. The estimated *R*_x_ ≈ (19.8 ± 2.3)/s of GM is, similarly to WM, lower than literature values that are based on a constrained MT model ((40 ± 1)/s (Stanisz et al., 2005)).

The estimated $T_{2}^{f}\approx (90.8\pm 6.4)\text{ms}$ exhibits slight deviations from literature values ((99 ± 7)ms), as does $T_{2}^{s}\approx (11.4\pm 1.1)\mu \text{s}$ in comparison to (9.1 ± 0.2)μs as estimated by Stanisz et al. (2005).

### 3.4. *In vivo* imaging of individuals with MS

#### 3.4.1. MS lesions

**Figure 6:**
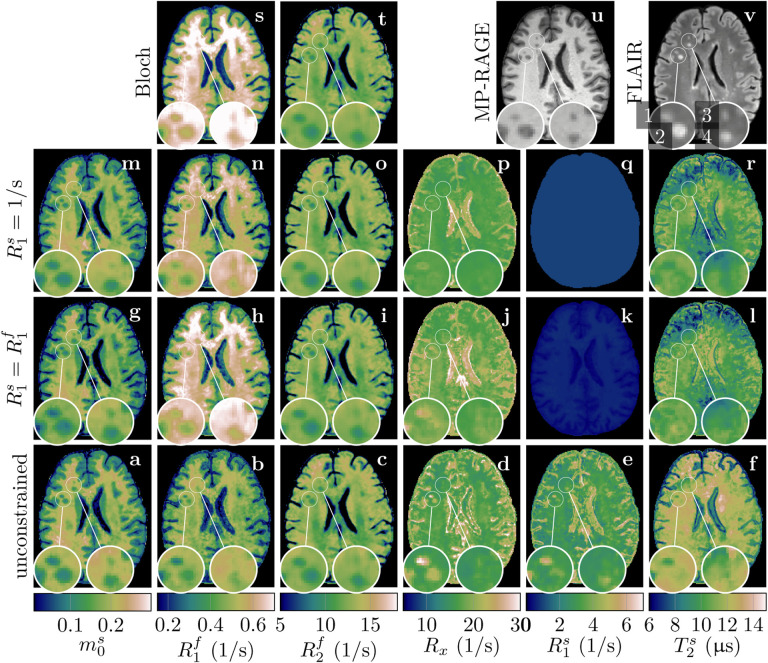
Quantitative MT maps of an individual with MS fitted with the proposed unconstrained MT model (**a-f**), MT model constrained by $R_{1}^{s}=R_{1}^{s}$ (**g**-**l**) and $R_{1}^{s}=1/\text{s}$ (**m**-**r**), and the Bloch model (**s,t**). Note that **k** is a replica of **h** on a different color scale. MP-RAGE (**u**) and FLAIR (**v**) scans are provided for reference. The magnifications highlight four lesions (labeled in **v**) that have similar appearances on the FLAIR and MP-RAGE and reveal heterogeneity on the qMT maps, heterogeneity that is most pronounced in the unconstrained qMT maps.

Fig. 6 shows a transverse slice through an MS patient’s brain, which contains several MS lesions. In the four highlighted lesions, but also in lesions throughout all 4 individuals with MS, we find a substantial reduction of $m_{0}^{s}$ consistent with demyelination observed with histology. This finding appears the same for both the constrained and unconstrained MT models. The other qMT parameters reveal substantial differences between the four highlighted lesions, in particular when using the unconstrained MT model. For example, the exchange rate *R*_x_ is substantially increased in lesion 1 and, to lesser degree, in lesion 2, while lesions 3 and 4 show virtually no contrast to NAWM (Fig. 6d). This heterogeneity is much less pronounced in the constrained MT parameter maps (g-r), and even less visible in the Bloch-derived relaxation maps (s,t) or coregistered MP-RAGE (u) and FLAIR (v) images. In the constrained MT models, $R_{1}^{f}$ is rather uniformly reduced in lesions 1,2, and 4 (Fig. 6h,n), while this decrease varies between the three lesions when using the unconstrained MT model (b), and the varying decrease is accompanied by an increase in *R*_x_ (d) and $R_{1}^{s}$ (e).

#### 3.4.2. MS pathology in normal appearing white and gray matter

**Figure 7:**
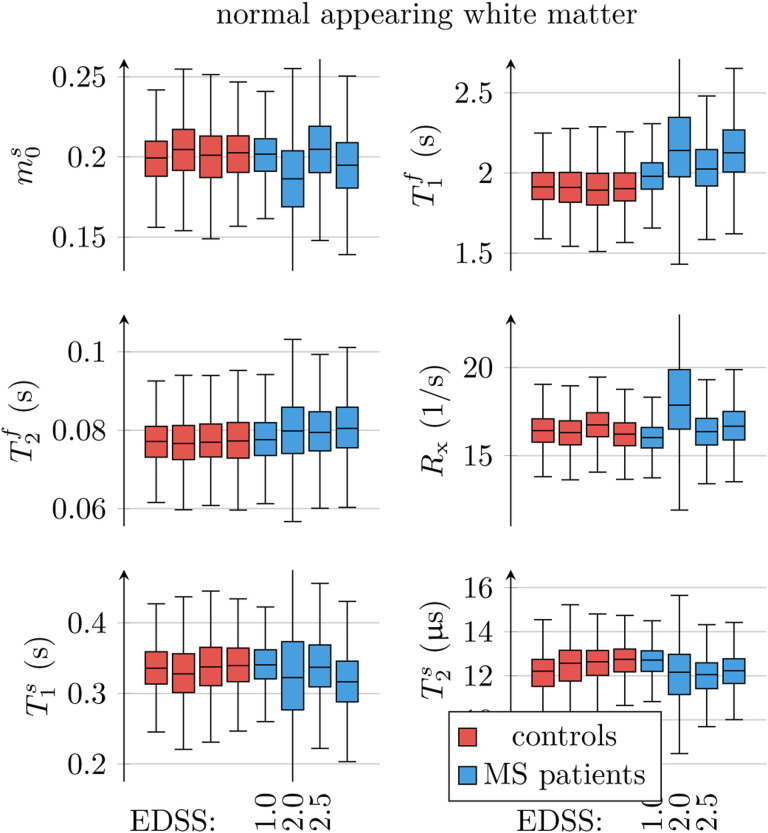
ROI analysis of the unconstrained qMT model’s parameters, pooled over all normal appearing white matter (NAWM) voxels in each of the 4 individuals with MS and the 4 controls. The patients’ score on the expanded disability status scale (EDSS) is provided where known.

Fig. 7 analyzes all unconstrained qMT parameters in an ROI that spans the entire NAWM. Visually, we observe the most distinct differences between individuals with MS and healthy controls in $T_{1}^{f}$. The median $T_{1}^{f}$ of each MS subject, averaged over all MS subjects, was 0.16s larger than in controls (*p* < 0.03). The median $T_{2}^{f}$ of each MS subject, averaged over all MS subjects, was 2.3ms larger than in controls (*p* < 0.03).

**Figure 8:**
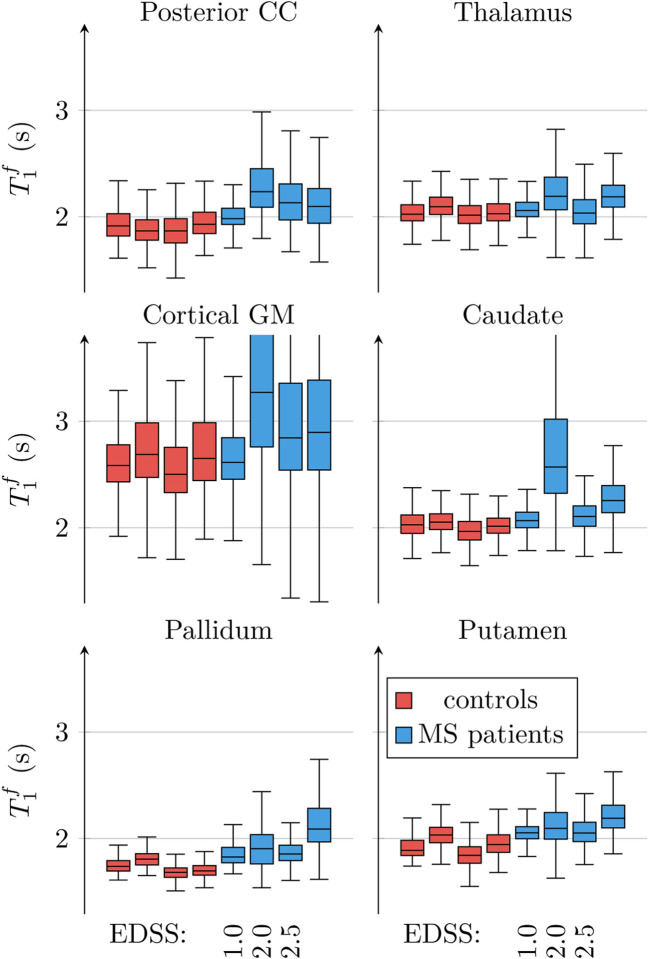
ROI analysis of the unconstrained model’s $T_{1}^{f}$. In addition to the analysis in Fig. 7 that analyzes the entire normal appearing white matter (NAWM), we analyze here selected gray matter (GM) regions and the posterior corpus callosum (CC). The patients’ score on the expanded disability status scale (EDSS) is provided where known.

Fig. 8 characterizes $T_{1}^{f}$ in several WM regions and GM structures. Visually, we observe abnormalities in the posterior corpus callosum (CC), the thalamus, the cortical GM, the caudate nucleus, the pallidum, and the putamen. Statistically, however, only the deviations in the posterior CC were significant (*p* < 0.03).

When analyzing all unconstrained qMT parameters for the ROIs listed in Tab. 2, we also found significant changes of *R*_x_ in the pallidum (*p* < 0.04) and of $T_{1}^{s}$ in the cortical GM (*p* < 0.01).

#### 3.4.3. Precision of qMT estimates

In order to gauge the precision of the qMT estimates, we calculated the CRB for each voxel in the transversal slice that is depicted in Fig. 6 and cuts through the brain of an individual with MS. In WM, we find good agreement between the optimized CRB values (Tab. 1) and the CRB for the estimated parameters, confirming that the optimization for a single point in parameter space was adequate. The biggest deviations to Tab. 1 can found in $m_{0}^{s}$, which is slightly higher for the in vivo WM estimates, and in $R_{1}^{f}$, which is slightly lower for the in vivo WM estimates.

In the cortical GM, we find CRB values similar to ones in WM for $m_{0}^{s}$, $R_{1}^{f}$, and $R_{2}^{f}$, and increased CRB values for *R*_x_, $R_{1}^{s}$, and $T_{2}^{s}$, which is expected due to the reduced $m_{0}^{s}$. This effect is also in line with increased noise levels reported in Tab. 2 and it is even more pronounced in CSF, where $m_{0}^{s}$ is close to zero. In the MS lesions, we see only slight increases in the CRB values $m_{0}^{s}$, $R_{1}^{f}$, and $R_{2}^{f}$, which gives confidence in the estimates of these parameters in lesions. The CRB values of *R*_x_, $R_{1}^{s}$ and $T_{2}^{s}$, on the other hand, do increase MS lesions, in particular in lesions 1 and 2 (cf. 9g). This is likely related to the pronounced decrease in $m_{0}^{s}$ in these lesions.

#### 3.4.4. Rapid qMT imaging

**Figure 10:**
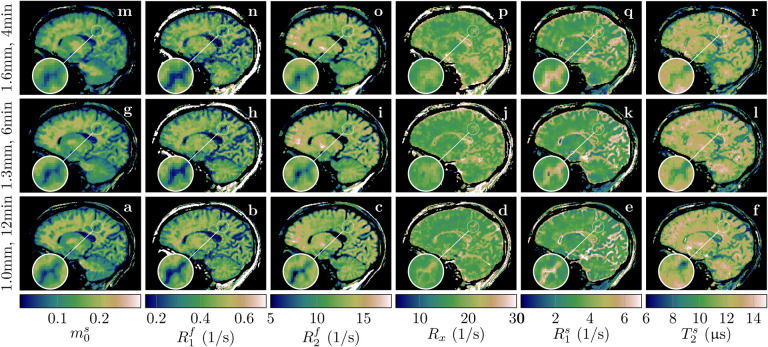
Quantitative MT maps of an individual with MS scanned with different (nominal) resolutions and different scan times. All scans have an isotropic resolution and were acquired with full brain coverage. The magnifications point at a cortical structure that visualizes the differences in resolution.

All data described thus far were acquired with 1mm isotropic resolution and 36min scan time. To gauge the potential of our qMT approach for more clinically-feasible scan times, we scanned an individual with MS with different resolutions and scan times. With 1mm isotropic resolution and 12min scan time, we observe overall good image quality despite slightly increased blurring compared to the 36min scan (cf. the cerebellum in Fig. 4f to the one in Fig. 10a). With 1.3mm isotropic nominal resolution and 6min scan time, we observe similar image quality besides the reduced resolution, and the same is true for 1.6mm isotropic in 4min.

## 4. Discussion

A major goal of this paper is to demonstrate the feasibility of unconstrained quantitative magnetization transfer imaging using a hybrid-state pulse sequence. We were able to quantify all 9 model parameters in vivo with a nominal resolution of 1mm isotropic and whole brain coverage using a 36 min scan. To the best of our knowledge, the presented maps are the first voxel-wise fits with an unconstrained MT model. We provide average parameter estimates of healthy brain tissue and these estimates confirm previous WM estimates with this model (Helms and Hagberg, 2009; Gelderen et al., 2016; Manning et al., 2021; Samsonov and Field, 2021) and provide estimates for additional smaller brain structures (Tab. 2).

Our data also confirms the previously reported differences to estimates with constrained MT models, most prominently the substantial differences between the longitudinal relaxation times of the free and the semi-solid spin pool (Fig. 5). This finding has important implications for for our understanding of longitudinal relaxation in biological tissue: two-pool MT models generally describe a bi-exponential longitudinal relaxation, which can be analyzed by an eigendecomposition (Gochberg and Gore, 2003). When assuming $R_{1}≔R_{1}^{s}=R_{1}^{f}$, the smaller (in the absolute value) eigenvalue is equal to *R*_1_ (Yarnykh, 2012). This implies that MT is an effect that primarily happens right after a separation of the two pools’ longitudinal magnetization, e.g., by saturating the semi-solid spin pool (Wolff and Balaban, 1989; Henkelman et al., 1993) or by selectively inverting the free pool (Gochberg and Gore, 2003). Once the two pools approach each other (which happens at the time scale *T*_x_ = 1*/R*_x_ ≈ 50ms), they decay mono-exponentially with $R_{1}≔R_{1}^{s}=R_{1}^{f}$, i.e. the semi-solid spin pool does not affect the relaxation of the free pool anymore.

An eigendecomposition of the unconstrained MT model also has two distinct eigenvalues (Gochberg and Gore, 2003) and we can consider the smaller eigenvalue an apparent relaxation rate $R_{1}^{a}$. A Taylor expansion of the apparent relaxation rate at $R_{1}^{s}=R_{1}^{f}$ facilitates a comparison to the above described constrained model:

(2) $$R_{1}^{a}\approx R_{1}^{f}+m_{0}^{s}\left(R_{1}^{s}-R_{1}^{f}\right)-\frac{m_{0}^{s}\left(1-m_{0}^{s}\right)\cdot {\left(R_{1}^{s}-R_{1}^{f}\right)}^{2}}{R_{\text{x}}}.$$

The linear correction term reveals that the apparent relaxation rate depends on the macromolecular pool size in addition to the two relaxation rates and higher order terms additionally depend on the exchange rate *R*_x_. Given $R_{1}^{s}\gg R_{1}^{f}$, as our data suggests for brain tissue, the apparent relaxation rate, thus, depends on all MT parameters and, given $R_{1}^{a}\gg R_{1}^{f}$, we conclude that magnetization transfer is an inherent driver of longitudinal relaxation.

Inserting WM estimates of the qMT parameters (Tab. 2) into Eq. (2) results in $T_{1}^{a}=1/R_{1}^{a}\approx 1.03\text{s}$, which is in line with mono-exponential estimates reported in the literature $T_{1}^{a}\approx 1.084\text{s}$ (Stanisz et al., 2005)). This concordance is expected for inversion recovery experiments, if either both spin pools are inverted by a short RF-pulse $\left(T_{\text{RF}}\ll T_{2}^{s}\right)$ or if all inversion times fulfil *T*_I_ ≫ 1/*R*_x_. Our pulse sequence does not fulfill either of these conditions, which explains the deviating $T_{1}^{a}\approx 1.429\text{s}$ when fitting a mono-exponential model to our data.

The central role of MT in longitudinal relaxation is further illustrated by the concordance between the pronounced GM/WM contrast in *T*_1_-weighted images (e.g. Fig. 4c,d) and in $m_{0}^{s}$ (Fig. 4e,f), which is more pronounced than in the other qMT parameters. Koenig et al. (1990) pointed at myelin as the primary source of GM/WM contrast in *T*_1_-weighted MRI and our work refines this well-established finding by suggesting MT is the primary mechanism that generates this contrast. However, we also observe a noteworthy GM/WM contrast in $T_{1}^{f}$ and this spatial distribution suggests that myelin facilitates regular longitudinal relaxation of the free spin pool on top of MT, possibly, by direct interactions between the water protons and the local magnetic field of the proteins and lipids (Gossuin et al., 2000).

$T_{1}^{f}$ of the globus pallidus is shorter compared all other ROIs that were analyzed in this study (Tab. 2). Since the globus pallidus is known to accumulate iron in the form of ferritin, this suggests a subtle sensitivity of $T_{1}^{f}$ to iron, which is in line with the reports of Vymazal et al. (1999) and Samsonov and Field (2021). However, our data suggests that $T_{1}^{f}$ it is not specific to iron.

For the transversal relaxation time $T_{2}^{f}$, we find a more pronounced shortening in the globus pallidus. This suggests that $T_{2}^{f}$ is more sensitive and more specific to iron than $T_{1}^{f}$, which is in line with previous findings by Schenker et al. (1993); Vymazal et al. (1999); Gossuin et al. (2000).

We note that our WM estimates of $T_{2}^{f}$ deviate from previous estimates (Stanisz et al., 2005). A possible explanation is that our model neglects contributions from myelin water (MW). MW is water that is trapped between the myelin sheaths and has a characteristic $T_{2}^{\text{MW}}\approx 10\text{ms}$ (Mackay et al., 1994). It exchanges magnetization with myelin’s macromolecular pool as well as the larger intra-/extra-axonal water pool, where the former exchange is faster than the latter (Stanisz et al., 1999; Manning et al., 2021). A saturation of the semi-solid pool pool could, thus, result in a saturation of the MW pool and ultimately to its suppression. An estimate of the apparent $T_{2}^{f}$, which comprises both the intra-/extra-axonal water pool and MW pool would, thus, be dominated by the former, while a CPMG sequence that starts from thermal equilibrium has more pronounced contributions of the MW pool, resulting in shorter effective relaxation times. This effect could explain the observed differences in the apparent relaxation times, but a more detailed analysis is needed for a thorough understanding of these deviations, which is beyond of the scope of the current report.

Fig. 6u highlights four MS lesions with a hypointense appearance in the MR-RAGE. Our data suggests that this hypointensity is foremost driven by a reduction of $m_{0}^{s}$, which was found for all examined lesions. In contrast, we find that changes in mono-exponential *T*_1_-estimates in NAWM are primarily driven by $T_{1}^{f}$ (Fig. 5). This juxtaposition of different sources of *T*_1_-contrast changes or changes in the apparent *T*_1_ once again highlights the complexity of longitudinal relaxation in biological tissue. It also highlights the increased specificity of qMT, in particular, unconstrained qMT compared to mono-exponential relaxation time mapping and clinical contrasts. This increased specificity was also demonstrated in *R*_x_ (Fig. 6d), where we observe elevated values in some, but not all lesions (lesions 1–2 vs. 3–4). Such pathological changes stand in contrast to the relatively smaller GM/WM contrast that we observe in *R*_x_ in line with previous reports (Yarnykh, 2012). We also find heterogeneity in the qMT parameters between MS lesions, which is consistent with known heterogeneity in MS lesion pathology, where there are varying degrees of remyelination, axonal damage, inflammation, and gliosis in individual lesions. Unconstrained qMT could potentially improve individual lesion characterization, disease staging, and the prediction of MS progression. Future work will include a quantitative analysis of the unconstrained qMT parameters in MS lesions.

Another goal of this paper was to gauge the sensitivity of unconstrained qMT to subtle changes in normal appearing WM and GM that are not easily detectable with established (contrast based) clinical sequences. We observed statistically significant deviations of $T_{1}^{f}$ between individuals with MS and healthy controls, in particular, in the NAWM, which is in line with previous studies that performed mono-exponential *T*_1_-mapping (Vrenken et al., 2006a,c,b). Further, we found statistically significant deviations of $T_{1}^{f}$ in subcortical GM structures. An analysis of NAMW in individuals with MS always bears the risk of contaminating the results with an incomplete exclusion of MS lesions or by voxels close to lesions. However, we have two reasons that make us believe that the observed changes in $R_{1}^{f}$ are not driven by lesions and their surrounding tissue: first, in lesions we observe predominantly changes in $m_{0}^{s}$, while $m_{0}^{s}$ changes in NAWM are much less pronounced. Second, Vrenken et al. (2006b) demonstrated that the magnetization transfer ratio in NAWM changes with the distance to an MS lesion, but their mono-exponential *T*_1_ estimates do not. However, larger studies are needed to confirm this result.

The main engineering goal of this work was the removal of model constraints. Our secondary focus was a voxel-wise fit at a clinically-established spatial resolution and we were indeed able to extract unconstrained qMT maps with 1mm, 1.3mm, and 1.6mm isotropic nominal resolution from 12min, 6min, and 4min scans, respectively. However, we do observe a subtle blurring in our qMT maps in comparison to the MP-RAGE. The most likely cause is the smaller k-space coverage of the koosh-ball trajectory in comparison to a Cartesian trajectory: the koosh-ball trajectory with a nominal resolution of 1mm samples only the inner sphere of the 1mm k-space cube, similar to *elliptical scanning*, while the MP-RAGE samples the entire cube. Undersampling, the regularized reconstruction, and incomplete motion correction might cause additional blurring. The source of and solutions to this blurring will be the subject of future research. Our ongoing work also includes efforts for further scan time reductions. To this end, we aim to replace the current RF-pattern, which is a concatenation of separate optimizations, with a joint optimization of the entire scan. Further, we are exploring more efficient k-space trajectories. Last, we hope that studies with the current pulse sequence will help with the identification of the clinically most promising parameters. This information can then be fed back to our numerical optimization framework to optimize pulse sequences for more efficient estimation of these parameters. CRB-based optimizations allow for such specializations without imposing constraints on the parameters. If further scan time reductions are needed, it is also plausible to adopt the approach of Yarnykh (2012) by constraining parameters that appear rather stable throughout the brain and in between subjects, such as $R_{1}^{s}$ and/or $T_{2}^{s}$. This approach would require careful validations similar to previous reports Yarnykh (2012).

## 5. Conclusion

Our work builds on the work of Helms and Hagberg (2009); Gelderen et al. (2016); Manning et al. (2021); Samsonov and Field (2021), who pioneered unconstrained fits with Henkelman’s two-pool magnetization transfer model. By utilizing the encoding power of the hybrid state (Assländer et al., 2019b), we were able to improve the sensitivity of MT data to the model’s parameters, which allowed us to fit the unconstrained MT model to each voxel separately at 1mm isotropic nominal resolution. Our results largely confirm previous findings, most notably the substantially different longitudinal relaxation times of the free and the semi-solid spin pools.

## Figures and Tables

**Figure 9:**
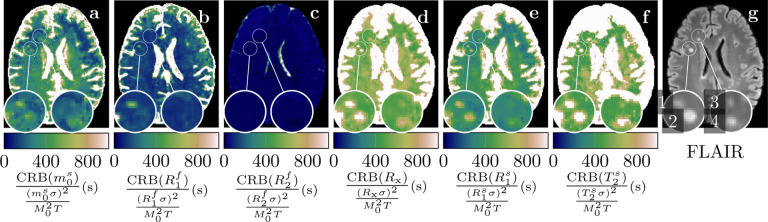
Cramér-Rao bound (CRB) of the in vivo parameter estimates shown in Fig. 6a–f, normalized to resemble the inverse squared SNR of the parameter estimates (cf. Tab. 1). For the CRB calculations, we used the unconstrained MT model and the corresponding estimates. The FLAIR image is provided for anatomical reference.

**Table 1:** Comparison of the Cramér-Rao bound (CRB) values (lower is better) between specialized optimizations for a single parameter, a concatenation of these 6 optimized RF-patterns, and a joint optimization of all parameters. The objective of each optimization is highlighted in gray. Each optimization treats all biophysical parameters, as well as *ω*_*z*_, *B*_1_, and the scaling factor *M*_0_ as unknowns, i.e. we assume an 9-parameter fit. The CRB values are normalized by the squared value of the parameter, the squared magnetization *M*_0_, and the noise variance of the time series in a voxel *σ*^2^, i.e. they reflect the inverse squared signal-to-noise ratio for a unit signal-noise variance. Further, they are normalized by the (simulated) scan time *T*, allowing for a fair comparison of the concatenated and the other patterns.

optimized for	$m_{0}^{s}$	$R_{1}^{f}$	$R_{1}^{f}$	*R*_x_	$R_{1}^{s}$	$T_{2}^{s}$	concat.	joint
$\text{CRB}\left(m_{0}^{s}\right)\cdot M_{0}^{2}/{\left(m_{0}^{s}\sigma \right)}^{2}\cdot T(\text{s})$	47	3837	23397	1576	19593	2169	99	119
$\text{CRB}\left(R_{1}^{f}\right)\cdot M_{0}^{2}/{\left(R_{1}^{f}\sigma \right)}^{2}\cdot T(\text{s})$	2649	91	6564	2608	1147	1674	237	427
$\text{CRB}\left(R_{2}^{f}\right)\cdot M_{0}^{2}/{\left(R_{2}^{f}\sigma \right)}^{2}\cdot T(\text{s})$	969	493	15	473	652	70	45	172
$\text{CRB}\left(R_{\text{x}}\right)\cdot M_{0}^{2}/{\left(R_{\text{x}}\sigma \right)}^{2}\cdot T(\text{s})$	21466	17598	52736	185	50744	15552	449	705
$\text{CRB}\left(R_{1}^{s}\right)\cdot M_{0}^{2}/{\left(R_{1}^{s}\sigma \right)}^{2}\cdot T\text{ (s)}$	24410	13581	20283	58802	264	6928	425	1263
$\text{CRB}\left(T_{2}^{s}\right)\cdot M_{0}^{2}/{\left(T_{2}^{s}\sigma \right)}^{2}\cdot T\text{ (s) }$	22270	3708	25178	10160	12876	203	646	717

**Table 2:** Region of interest (ROI) analysis in healthy controls. The ROIs were determined by segmenting the MP-RAGE images with the *FreeSurfer* software after co-registering it to the qMT coefficient images. The values represent the mean and standard deviation of all voxels from 4 healthy subjects.

	$m_{0}^{s}$	$T_{1}^{f}\;(\text{s})$	$T_{2}^{f}\;(\text{ms})$	*R*_x_ (1/s)	$T_{1}^{s}\;(\text{s})$	$T_{2}^{s}\;(\mu \text{s})$
entire WM	0.200 ± 0.021	1.92 ± 0.17	77.6 ± 7.0	16.5 ± 1.2	0.337 ± 0.042	12.4 ± 1.0
anterior CC	0.223 ± 0.031	1.88 ± 0.28	71.8 ± 5.1	17.1 ± 1.8	0.338 ± 0.047	13.58 ± 0.93
posterior CC	0.222 ± 0.032	1.94 ± 0.25	78.4 ± 5.0	17.0 ± 1.8	0.359 ± 0.059	12.60 ± 0.61
cortical GM	0.091 ± 0.019	2.70 ± 0.46	84.3 ± 7.8	20.5 ± 3.2	0.315 ± 0.096	11.7 ± 1.3
Caudate	0.106 v 0.016	2.04 ± 0.15	75.0 ± 3.7	19.3 ± 2.5	0.376 ± 0.080	12.85 ± 0.57
Putamen	0.122 ± 0.016	1.95 ± 0.16	69.1 ± 4.3	18.0 ± 1.4	0.373 ± 0.050	13.16 ± 0.69
Pallidum	0.158 ± 0.015	1.75 ± 0.12	61.7 ± 5.3	19.1 ± 1.4	0.361 ± 0.041	13.61 ± 0.98
Thalamus	0.156 ± 0.024	2.08 ± 0.23	72.6 ± 5.4	17.6 ± 2.1	0.400 ± 0.072	12.37 ± 0.87

## Data Availability

In order to promote reproducibility, we provide the latest version (v0.8.0, DOI:10.5281/zenodo.7433494) of the sequence optimization and signal simulation source code on https://github.com/JakobAsslaender/MRIgeneralizedBloch.jl. They are written in the open-source language Julia and we registered the package ”MRIgeneralizedBloch.jl” with Julia’s package manager. The documentation of the package along with tutorials can be found on https://JakobAsslaender.github.io/MRIgeneralizedBloch.jl. The tutorials render the code in HTML format with interactive figures and link to Jupyter notebooks that can be launched in *binder*, enabling an interactive learning in a browser without any local installations. Further, we provide the source code of the image reconstruction package on https://github.com/JakobAsslaender/MRFingerprintingRecon.jl, which is also written in Julia. For the here-presented data, we used v0.3.5. Last, we made the qMT maps of all subjects available on https://doi.org/10.5281/zenodo.7492581.

## abbreviations:

WM: white matter
GM: gray matter
MS: multiple sclerosis
(q)MT: quantitative) magnetization transfer
CRB: Cramér-Rao bound
BSA: bovine serum albumin
NN: neural network
ROI: region of interest
NAWM: normal appearing white matter
EDSS: expanded disability status scale
CC: corpus callosum
MP-RAGE: magnetization-prepared rapid gradient-echo
FLAIR: fluid attenuated inversion recovery
MW: myelin water
