# Supplementary material for: Unconstrained quantitative magnetization transfer imaging: disentangling T1 of the free and semi-solid spin pools
Source: ArXiv. 2024 Apr 1:arXiv:2301.08394v3. Originally published 2023 Jan 20. Preprint. [Version 3] (PMC9882584)
Supplement: Supplement 1 [file NIHPP2301.08394v3-supplement-1.pdf]

Supporting Information for: Unconstrained quantitative magnetization transfer imaging: disentangling T_1 of the free and semi-solid spin pools

Jakob Assländer[1,a,b], Andrew Mao[a,b,c], Elisa Marchetto[a,b], Erin S Beck[d], Francesco La Rosa[d], Robert W Charlson[e], Timothy M Shepherd[a], Sebastian Flassbeck[a,b]

[a] *Center for Biomedical Imaging, Dept. of Radiology, New York University School of Medicine, 650 1st Avenue, New York, 10016, NY, USA*
[b] *Center for Advanced Imaging Innovation and Research (CAI2R), Dept. of Radiology, New York University School of Medicine, 650 1st Avenue, New York, 10016, NY, USA*
[c] *Vilcek Institute of Graduate Biomedical Sciences, New York University School of Medicine, 550 1st Avenue, New York, 10016, NY, USA*
[d] *Corinne Goldsmith Dickinson Center for Multiple Sclerosis, Department of Neurology, Icahn School of Medicine at Mount Sinai, 5 East 98th Street, New York, 10029, NY, USA*
[e] *Department of Neurology, New York University School of Medicine, 240 E 38th Street, New York, 10016, NY, USA*

arXiv:2301.08394v3 [physics.med-ph] 1 Apr 2024

S1. Numerical optimizations of the pulse train

The numerical optimizations of the pulse train resulted in smooth flip angle and T_{RF} patterns. Fig. S1 sketches the RF patterns that were optimized for m_0^s and R_1^f, respectively, along with the evoked spin trajectories. We observe distinct patterns for the different optimization objectives (Fig. S1) that correspond to distinct CRB values (Tab. S1). For example, optimizing for m_0^s resulted in a normalized CRB of 47s for m_0^s and of 3837s for R_1^f, while the optimization for R_1^f resulted in a normalized CRB of 2649s for m_0^s and of 91s for R_1^f. This difference in CRB values is in line with the noise levels in scans with each of the two RF patterns: the optimization for m_0^s results in a comparably low noise level in m_0^s (Fig. S1e) and a comparably high noise level in R_1^f (f), while the optimization for R_1^f results in the opposite noise characteristics (l vs. m).

As the optimizations aim to disentangle the effect of 9 overall parameters, the resulting spin trajectories are difficult to interpret. Nonetheless, we can discern some features: for example, the m_0^s-optimized pattern starts with near-zero flip angles after the inversion pulse, which provokes a bi-exponential inversion recovery of the longitudinal magnetization (circular magnification in Fig. S1c) that encodes m_0^s similar to the *SIR* method proposed by Gochberg and Gore (2003). The rectangular magnification in Fig. S1 highlights a section of the spin dynamics in which large flip angles, combined with short T_{RF}, saturate the semi-solid spin pool, which resembles the encoding mechanism proposed by Gloor et al. (2008). This saturation maximizes the difference between pools, and this encoding mechanism is not as pronounced in the spin trajectory evoked by the R_1^f-optimized pattern, where the semi-solid spin pool plays a subordinate role.

Concatenating the 6 individual optimizations for m_0^s, R_1^f, R_2^f, R_{x}, R_1^s, and T_2^s, respectively, increases the CRB values (normalized by the scan time) compared to the optimized CRB value of each specialized RF-pattern, but provides overall low CRB values for all six parameters that also results in high-quality parameter maps (Fig. S1o-q). Interestingly, these CRB values are consistently lower than a joint optimization for all parameters. We note that the latter has to encode all parameters in a single 4s-long cycle. At the same time, the former utilizes 6 distinct RF patterns and has, thus, more degrees of freedom to induce different spin dynamics. Given this result, we performed all experiments with concatenated scans that utilize the 6 individual optimizations.

S2. Noise analysis

In order to relate the Cramèr-Rao bound to noise levels in the parameter maps, we measured a BSA phantom (cf. Section 3.3 in the main paper) 25 times for 12min with 1.24mm effective resolution. We reconstructed each dataset with the pipeline described in Section 3.5, but without the locally-low-rank regularization to minimize distortions of the noise statistics. We calculated the parameter-to-noise ratio (PNR) voxel-by-voxel by dividing each parameter's estimate by the standard deviation over the 25 repetitions. Fig. S2 visualizes the PNR-variability between different voxels of the same sample as box plots.

For comparison, we estimated the anticipated PNR based on the Cramèr-Rao bound. For each BSA concentration, we used the median parameter estimates and the

[1]corresponding author: Jakob Assländer, Center for Biomedical Imaging, Department of Radiology, New York University School of Medicine, 650 1st Avenue, New York, NY 10016, USA. jakob.asslaender@nyumc.org

[2]abbreviations: WM: white matter, GM: gray matter, MS: multiple sclerosis, (q)MT: (quantitative) magnetization transfer, CRB: Cramér-Rao bound, BSA: bovine serum albumin, NN: neural network, ROI: region of interest, NAWM: normal-appearing white matter, EDSS: expanded disability status scale, CC: corpus callosum, MP-RAGE: magnetization-prepared rapid gradient-echo, FLAIR: fluid-attenuated inversion recovery, MW: myelin water

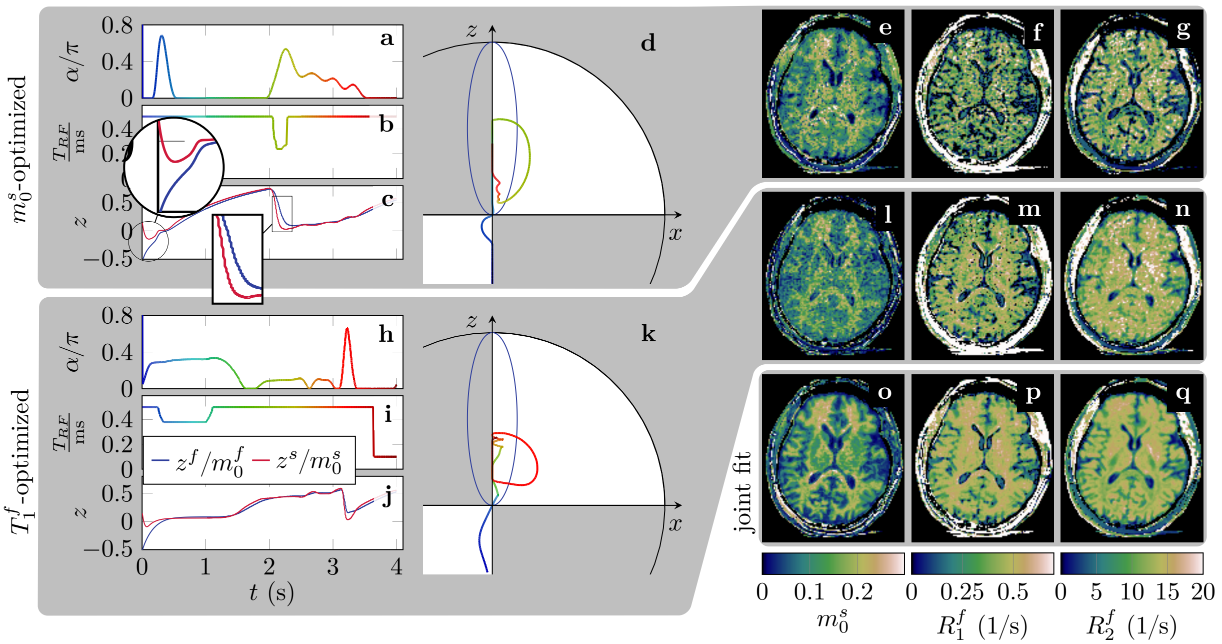

Figure S1: Optimized RF-pulse trains, evoked spin dynamics, and corresponding in vivo parameter maps. **a,h** The flip angle α and **b,i** the pulse duration T_{RF} control the spin dynamics. **c,j** The normalized z-magnetization of the two pools. The spherical and rectangular magnifications in (c) highlight segments that utilize a bi-exponential inversion-recovery (Gochberg and Gore, 2003) and saturation of the semi-solid spin pool (Gloor et al., 2008), respectively, which encode the semi-solid pool size m_0^s. **d,k** The dynamics of the free pool on the Bloch sphere with the steady-state ellipse in blue. **e-g** m_0^s and the relaxation times of the free pool T_1^f and T_2^f that were estimated from a 6min scan with an m_0^s-optimized RF-pattern in comparison to **l-n** a pattern that was optimized for T_1^f and **o-q** a joint fit of 6 measurements with RF-patterns that were optimized for different qMT parameters (36min scan time). We note that parameter maps are shown here to illustrate the trend, but we cannot expect a one-to-one match with the CRB values shown in Tab. S1 as the CRB does not directly predict the noise variance for most practical estimators, but we rather use it as a proxy for the conditioning of the fitting problem (cf. Section 3.2 in the main paper).

median noise variance in the coefficient images to calculate the CRB. The CRB-based PNR is then given by the median parameter estimate divided by the square root of the CRB (x-markers in Fig. S2). Comparing the experimental to the CRB-based PNR, we observe overall good correspondence. For virtually all parameters and samples, the CRB-based PNR lies within 1.5x the interquartile range, indicated by the whiskers. The residual discrepancy can be explained by the CRB's role as a mere proxy for the PNR: the CRB is achieved only for an unbiased and fully efficient estimator. Since neither non-linear-least square fitting nor the here-used neural-network fitting is strictly unbiased (Newey and McFadden, 1994; Wu, 1981), the CRB can only serve as a proxy and Fig. S2 demonstrates sufficient concordance to render the CRB a useful metric for parameter estimation (cf. Section 3.2 in the main paper).

To understand the effect of the employed locally-low-rank regularization on the parameter estimates, we repeated the phantom reconstructions with our default pipeline. Fig. S3 compares the median estimates and their variability throughout each sample and the 25 repetitions (pooled together). The median values overall align well between the two reconstructions and we observe reduced variability in the estimates when utilizing regularization.

S2.1. Precision of in vivo qMT estimates

In order to gauge the precision of the qMT estimates, we calculated the CRB for each voxel in the transversal slice of an individual with MS (Fig. S6). In WM, we find good agreement between the optimized CRB values (Tab. S1) and the CRB for the estimated parameters, confirming that the optimization for a single point in parameter space was adequate. The biggest deviations to Tab. S1 can be found in m_0^s, which is slightly higher for the in vivo WM estimates, and in R_1^f, which is slightly lower for the in vivo WM estimates.

In the cortical GM, we find CRB values similar to the ones in WM for m_0^s, R_1^f, and R_2^f, and increased CRB values for R_{x}, R_1^s, and T_2^s, which is expected due to the reduced m_0^s. This effect is also in line with increased noise levels reported in Tab. 2 of the main paper and is even more pronounced in CSF, where m_0^s is close to zero. In the MS lesions, we see only slight increases in the CRB values m_0^s, R_1^f, and R_2^f, which gives confidence in the estimates of these parameters in lesions. The CRB values of R_{x}, R_1^s, and T_2^s, on the other hand, do increase MS lesions, in particular in lesions 1 and 2 (cf. Fig. S6g). This increase is likely related to the lesions' pronounced decrease in m_0^s.

optimized for	m_0^s	R_1^f	R_2^f	R_x	R_1^s	T_2^s	joint	concat.	concat.
$\mathrm{CRB}(m_0^s) \cdot M_0^2/(m_0^s\sigma)^2 \cdot T$ (s)	47	3837	23397	1576	19593	2169	119	99	98
$\mathrm{CRB}(R_1^f) \cdot M_0^2/(R_1^f\sigma)^2 \cdot T$ (s)	2649	91	6564	2608	1147	1674	427	237	119
$\mathrm{CRB}(R_2^f) \cdot M_0^2/(R_2^f\sigma)^2 \cdot T$ (s)	969	493	15	473	652	70	172	45	44
$\mathrm{CRB}(R_x) \cdot M_0^2/(R_x\sigma)^2 \cdot T$ (s)	21466	17598	52736	185	50744	15552	705	449	437
$\mathrm{CRB}(R_1^s) \cdot M_0^2/(R_1^s\sigma)^2 \cdot T$ (s)	24410	13581	20283	58802	264	6928	1263	425	fixed
$\mathrm{CRB}(T_2^s) \cdot M_0^2/(T_2^s\sigma)^2 \cdot T$ (s)	22270	3708	25178	10160	12876	203	717	646	628

Table S1: Comparison of the Cramér-Rao bound (CRB) values (lower is better) between specialized optimizations for a single parameter, a concatenation of these 6 optimized RF patterns, and a joint optimization of all parameters. The objective of each optimization is highlighted in gray. The CRB values are normalized by the squared value of the parameter, the squared magnetization M_0, and the noise variance of the time series in a voxel σ^2, i.e., they reflect the inverse squared signal-to-noise ratio for a unit signal-noise variance. Further, they are normalized by the (simulated) scan time T, allowing for a fair comparison of the concatenated and the other patterns. Each optimization treats all biophysical parameters, as well as ω_z, B_1^+, and the scaling factor M_0 as unknowns, i.e., we assume a 9-parameter fit, with the exception of the last row that treats R_1^s as known for comparison.

	$m_0^{s,a}$	$T_1^{f,a}$ (s)	R_x^a (1/s)
entire WM	0.151 ± 0.022	0.941 ± 0.069	16.1 ± 1.2
ant. CC	0.174 ± 0.029	0.899 ± 0.065	15.8 ± 1.9
post. CC	0.173 ± 0.033	0.914 ± 0.074	15.9 ± 2.1
cort. GM	0.071 ± 0.051	1.62 ± 0.23	16.4 ± 3.4
Caudate	0.086 ± 0.019	1.389 ± 0.080	16.0 ± 2.5
Putamen	0.089 ± 0.015	1.271 ± 0.096	17.3 ± 2.0
Pallidum	0.125 ± 0.022	1.032 ± 0.049	18.3 ± 1.9
Thalamus	0.119 ± 0.024	1.23 ± 0.13	16.4 ± 2.1
Hippoc.	0.070 ± 0.023	1.62 ± 0.16	18.0 ± 3.1

Table S2: Region of interest (ROI) analysis of apparent qMT parameters (Eqs. (3)–(5)) in healthy controls. The ROIs were determined by segmenting the MP-RAGE images with the *FreeSurfer* software after co-registering it to the qMT coefficient images. The values represent the mean and standard deviation of all voxels from 4 healthy participants.

References

Gloor, M., Scheffler, K., Bieri, O., 2008. Quantitative magnetization transfer imaging using balanced SSFP. Magnetic Resonance in Medicine 60, 691–700. doi:10.1002/mrm.21705.

Gochberg, D.F., Gore, J.C., 2003. Quantitative imaging of magnetization transfer using an inversion recovery sequence. Magnetic Resonance in Medicine 49, 501–505. doi:10.1002/mrm.10386.

Hallgren, B., Sourander, P., 1958. The effect of age on the non-haemin iron in the human brain. Journal of Neurochemistry 3, 41–51. doi:10.1111/j.1471-4159.1958.tb12607.x.

Newey, W.K., McFadden, D., 1994. Chapter 36 Large sample estimation and hypothesis testing. Handbook of Econometrics 4, 2111–2245. doi:10.1016/s1573-4412(05)80005-4.

Wu, C.F., 1981. Asymptotic Theory of Nonlinear Least Squares Estimation. The Annals of Statistics 9, 501–513. doi:10.1214/aos/1176345455.

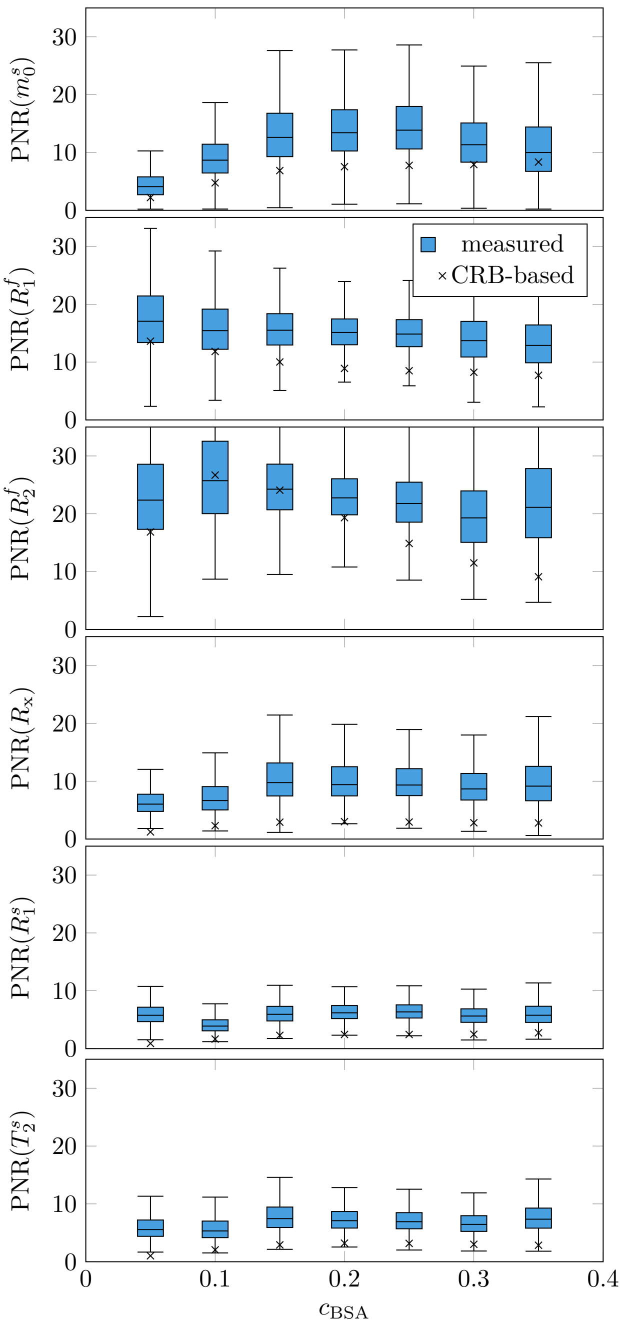

Figure S2: Parameter to noise ratio (PNR) of phantom scans at 2.9T. The PNR was measured voxel-by-voxel based on 25 repetitions of the same scan (reconstructed without any regularization) and the box plots show the inter-voxel variability of the estimated PNR. The x-markers identify the PNR as estimated by the median parameter estimates divided by the corresponding Cramèr-Rao bound (CRB).

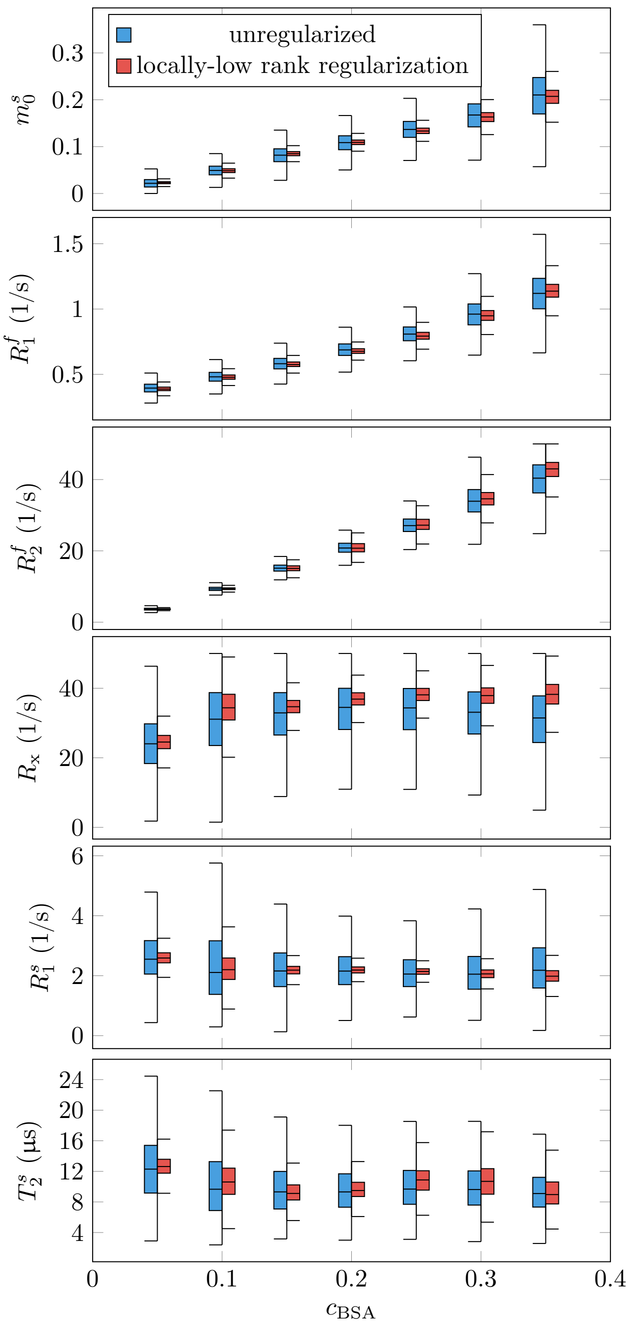

Figure S3: Comparison of parameter estimates between unregularized and regularized reconstructions at 2.9T. Each box plot analyzes the parameter estimates in a sample, pooled over all voxels in respective ROI and over 25 repetitions of the scan. The utilized regularization, including the regularization parameter, was identical to the reconstruction of the in vivo data shown in Fig. 8.

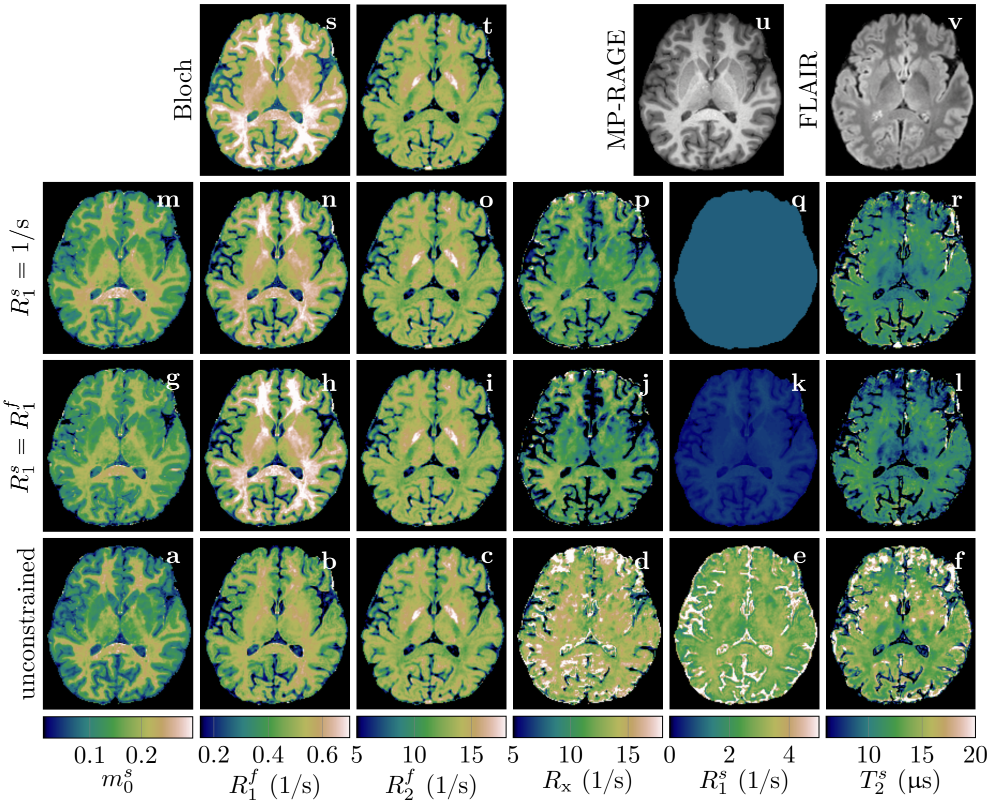

Figure S4: Quantitative MT maps of a healthy participant fitted with the proposed unconstrained MT model (**a-f**), MT model constrained by $R_1^s = R_1^s$ (**g-l**) and $R_1^s = 1/\mathrm{s}$ (**m-r**), and the Bloch model (**s,t**). The fits with each model are based on separately trained neural networks. Note that **k** is a replica of **h** on a different color scale. MP-RAGE (**u**) and FLAIR (**v**) scans are provided for reference.

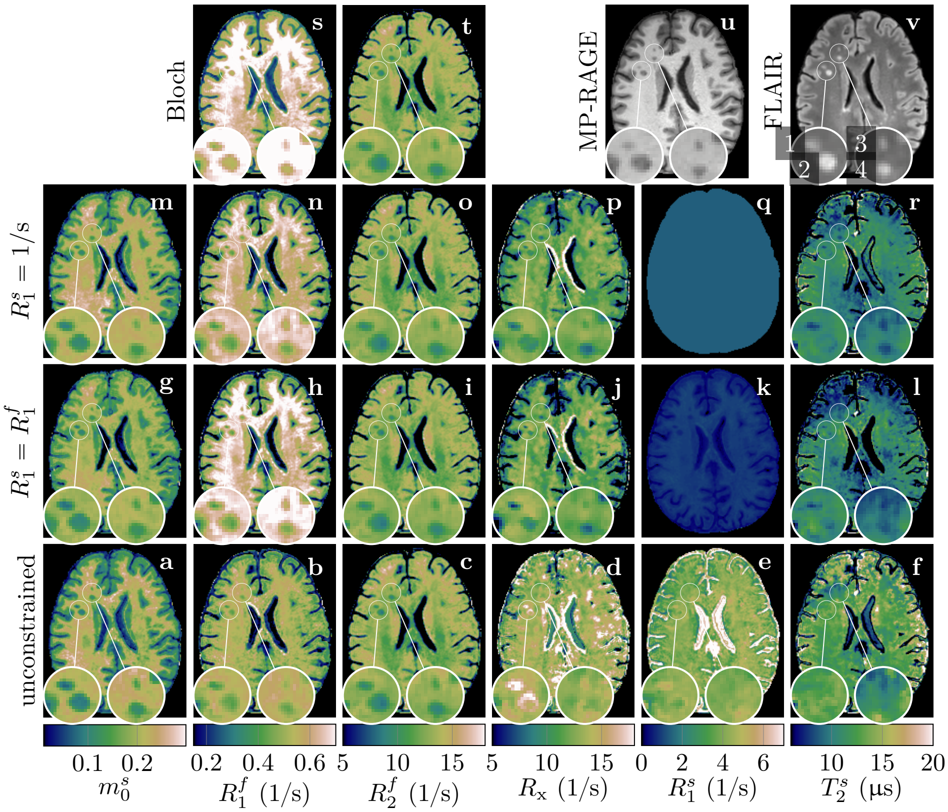

Figure S5: Quantitative MT maps of an individual with MS fitted with the proposed unconstrained MT model (**a-f**), MT model constrained by $R_1^s = R_1^f$ (**g-l**) and $R_1^s = 1/\text{s}$ (**m-r**), and the Bloch model (**s,t**). The fits with each model are based on separately trained neural networks. Note that **k** is a replica of **h** on a different color scale. MP-RAGE (**u**) and FLAIR (**v**) scans are provided for reference. The magnifications highlight four lesions (labeled in **v**) that have similar appearances on the FLAIR and MP-RAGE and reveal heterogeneity on the qMT maps, which is most pronounced in the unconstrained qMT maps.

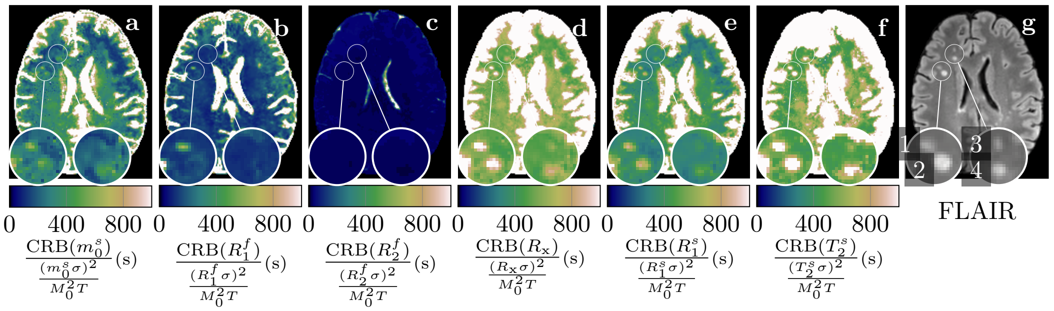

Figure S6: Cramér-Rao bound (CRB) of the in vivo parameter estimates shown in Fig. S5a-f, normalized to resemble the inverse squared SNR of the parameter estimates (cf. Tab. S1). We used the unconstrained MT model and the corresponding estimates for the CRB calculations. The FLAIR image is provided for anatomical reference.

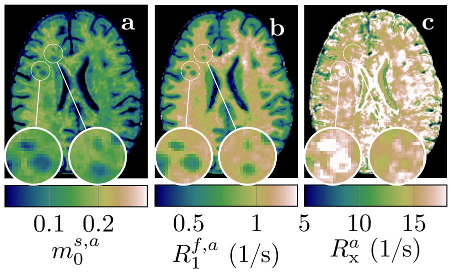

Figure S7: Apparent quantitative MT maps when assuming $T_1^s = T_1^f$ in a participant with MS. The maps were calculated voxel-wise with Eqs. (3)–(5) and based on the maps depicted in Fig. S5. Note the different color scale in $R_1^{f,a}$ compared to Fig. 3.

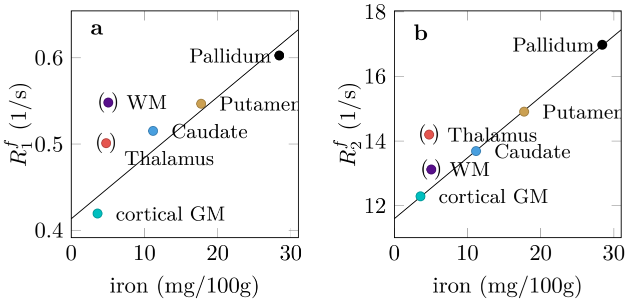

Figure S8: Relaxation rates, measured with our qMT approach, as a function of iron concentrations, taken from the literature (Hallgren and Sourander, 1958). **a:** Except for WM and the thalamus, which were excluded from the fit, the longitudinal relaxation rate $R_1^f = 1/T_1^f$ follows in good approximation a linear function ($R^2 = 0.94$). **b:** The transversal relaxation rate of the free pool $R_2^f = 1/T_2^f$ is even better described by a linear model ($R^2 = 0.9998$).

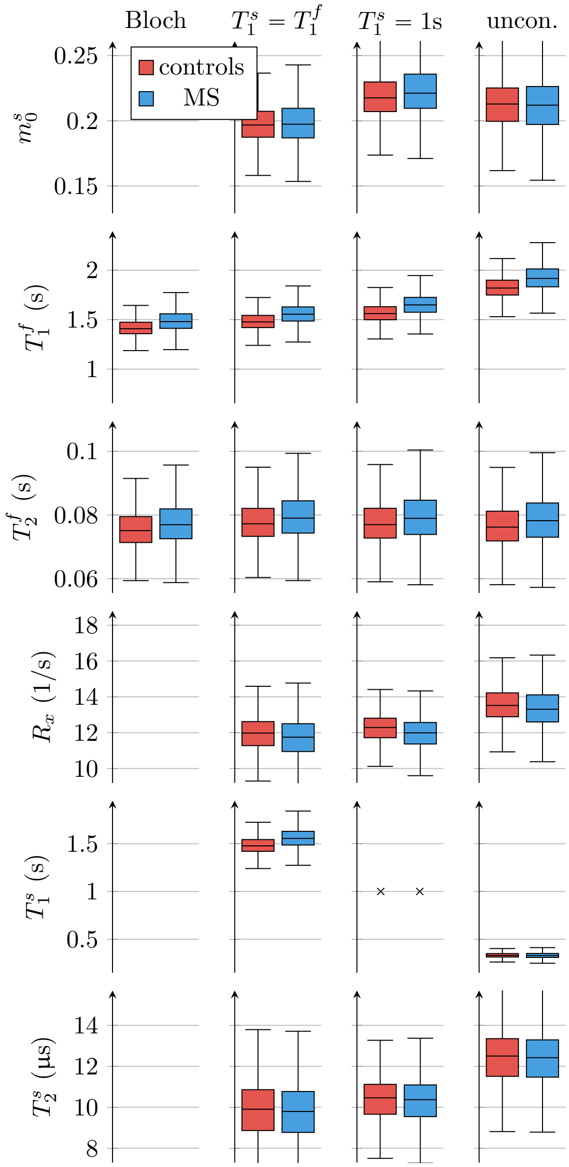

Figure S9: Comparison of the parameter estimates between a Bloch model, two traditional MT models that assume $T_1^s = T_1^f$ and $T_1^s = 1$s, respectively, and the proposed unconstrained MT model. This analysis pools all normal-appearing white matter voxels of 4 healthy participants and 4 individuals with MS, respectively. Note that the $T_1^s = T_1^f$ column depicts the same longitudinal relaxation time estimates twice to illustrate the differences to the unconstrained MT model.

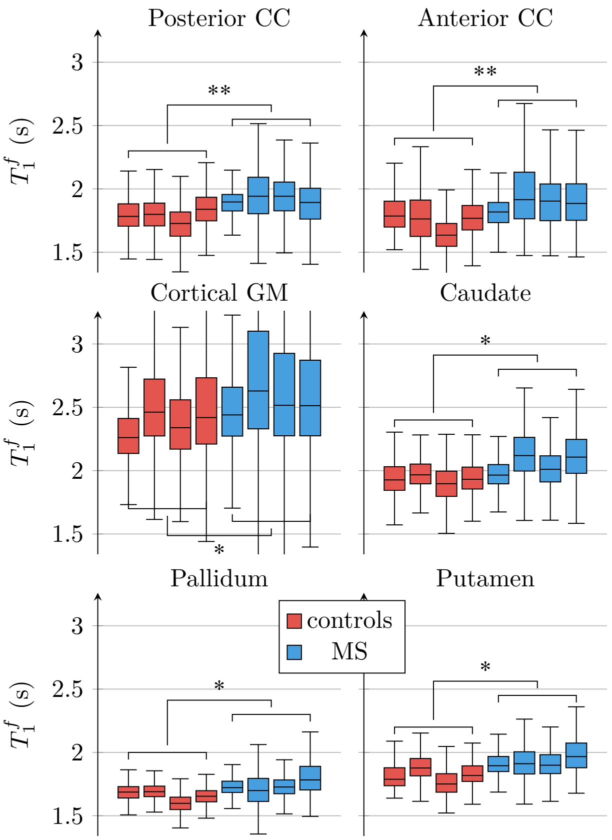

Figure S10: ROI analysis of the unconstrained model's T_1^f. For all six WM and GM ROIs, we found statistically significant differences between participants with MS and controls. The markers $*$ and $**$ indicate statistically significant differences at the $p < 0.05$ and $p < 0.01$ levels.
